# Obscure gastrointestinal bleeding: case report of ectopic pancreas associated with small bowel angiodysplasia

**DOI:** 10.1093/jscr/rjaf1019

**Published:** 2025-12-28

**Authors:** Fernando Girardi da Silva, Claudio Lucas Silva Cunha, Marina Capovilla Venturini Souza, Ana Luísa Costa Bezerra, Alexandre Foratto, Cesar Vanderlei Carmona, Talita Sansoni, Vânia Graner Silva Pinto, Thiago R A Calderan, Vitor Favali Kruger, Simone Reges Perales, Elcio Shiyoiti Hirano, Gustavo Pereira Fraga

**Affiliations:** Division of Trauma Surgery, University of Campinas (Unicamp), Rua Vital Brasil, 251, Cidade Universitária Zeferino Vaz, Campinas, SP 13083-887, Brazil; Division of Trauma Surgery, University of Campinas (Unicamp), Rua Vital Brasil, 251, Cidade Universitária Zeferino Vaz, Campinas, SP 13083-887, Brazil; Division of Trauma Surgery, University of Campinas (Unicamp), Rua Vital Brasil, 251, Cidade Universitária Zeferino Vaz, Campinas, SP 13083-887, Brazil; Division of Trauma Surgery, University of Campinas (Unicamp), Rua Vital Brasil, 251, Cidade Universitária Zeferino Vaz, Campinas, SP 13083-887, Brazil; Division of Trauma Surgery, University of Campinas (Unicamp), Rua Vital Brasil, 251, Cidade Universitária Zeferino Vaz, Campinas, SP 13083-887, Brazil; Division of Trauma Surgery, University of Campinas (Unicamp), Rua Vital Brasil, 251, Cidade Universitária Zeferino Vaz, Campinas, SP 13083-887, Brazil; Division of Trauma Surgery, University of Campinas (Unicamp), Rua Vital Brasil, 251, Cidade Universitária Zeferino Vaz, Campinas, SP 13083-887, Brazil; Division of Trauma Surgery, University of Campinas (Unicamp), Rua Vital Brasil, 251, Cidade Universitária Zeferino Vaz, Campinas, SP 13083-887, Brazil; Division of Trauma Surgery, University of Campinas (Unicamp), Rua Vital Brasil, 251, Cidade Universitária Zeferino Vaz, Campinas, SP 13083-887, Brazil; Division of Trauma Surgery, University of Campinas (Unicamp), Rua Vital Brasil, 251, Cidade Universitária Zeferino Vaz, Campinas, SP 13083-887, Brazil; Division of Trauma Surgery, University of Campinas (Unicamp), Rua Vital Brasil, 251, Cidade Universitária Zeferino Vaz, Campinas, SP 13083-887, Brazil; Division of Trauma Surgery, University of Campinas (Unicamp), Rua Vital Brasil, 251, Cidade Universitária Zeferino Vaz, Campinas, SP 13083-887, Brazil; Division of Trauma Surgery, University of Campinas (Unicamp), Rua Vital Brasil, 251, Cidade Universitária Zeferino Vaz, Campinas, SP 13083-887, Brazil

**Keywords:** ectopic pancreas, angiodysplasia, obscure gastrointestinal bleeding, jejunum

## Abstract

Obscure gastrointestinal bleeding (OGIB) is defined as bleeding not identified by standard endoscopy or colonoscopy and requires advanced diagnostic modalities. Ectopic pancreas is a rare congenital anomaly, usually asymptomatic but occasionally presenting with abdominal pain, obstruction, or gastrointestinal bleeding. We report a 23-year-old female admitted with melena and hemodynamic instability. Initial investigations were inconclusive. Persistent bleeding led to urgent laparotomy, which revealed jejunal nodular lesions. Segmental enterectomy was performed, with favorable recovery. Histology confirmed ectopic pancreatic tissue associated with jejunal angiodysplasia. This rare association highlights diagnostic and therapeutic challenges of OGIB and the importance of surgical intervention in unstable patients.

## Introduction

Obscure gastrointestinal bleeding (OGIB) occurs when the source is not identified by upper endoscopy or colonoscopy [1]. Investigation requieres capsule endoscopy, enteroscopy, or angiography, depending on severity. Common causes include Dieulafoy lesions, angiodysplasia, and small bowel tumors [2].

Ectopic pancreas (EP) is pancreatic tissue located outside its usual site, without continuity with the main gland. Its incidence ranges from 0.6% to 14% in autopsies [3]. It is most often found in the stomach or small intestine, though other sites have been described [4]. Clinical manifestations include abdominal pain, obstruction, perforation, or gastrointestinal bleeding [5].

We report a rare case of jejunal EP associated with angiodysplasia presenting as OGIB.

## Case report

A 23-year-old female presented with six days of melena and a recent episode of massive hematochezia. She had no comorbidities or relevant medication use. On admission, she was hypotensive (90/46 mmHg), tachycardic (116 bpm), with hemoglobin 6.7 g/dL. Initial management included endotracheal intubation and volemic replacemente [fluids and three units of packed red blood cells (PRBCs)].

**Figure 1:**
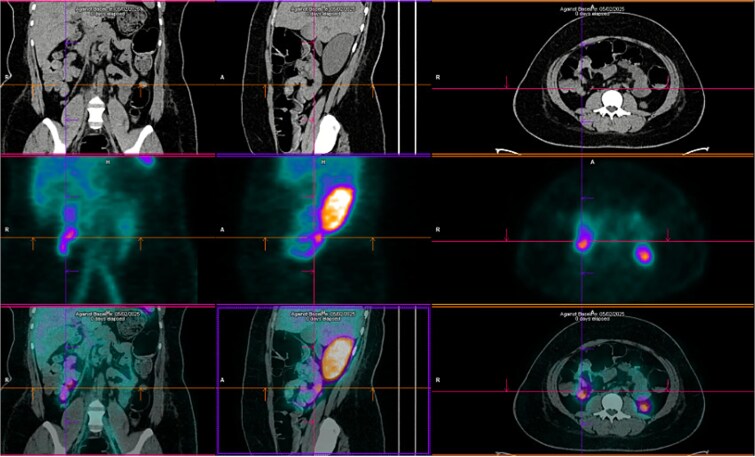
Technetium-labeled red blood cells scintigraphy showing two foci of active bleeding in the mesogastric and hypogastric regions.

**Figure 2:**
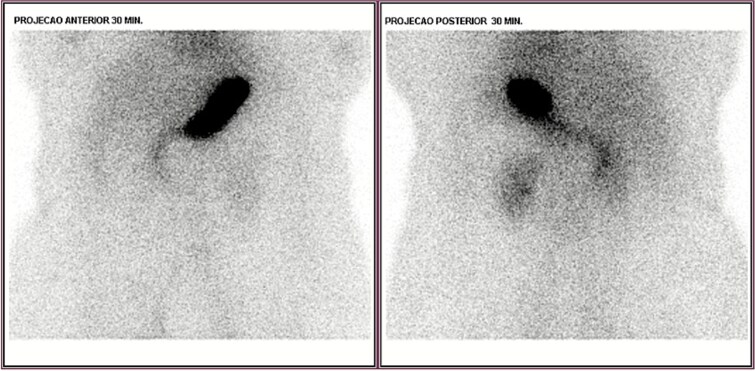
Negative scintigraphy for Meckel’s diverticulum.

Upper endoscopy, abdominal computed tomography (CT), and colonoscopy were inconclusive. CT angiography showed focal wall thickening in the proximal jejunum, 20 cm distal to the ligament of Treitz, with a subsegmental vascular branch. She was admitted to the intensive care unit (ICU), transfused with two additional PRBCs, and extubated on day two. Despite this, she had intermittent melena. Red blood cell scintigraphy demonstrated two bleeding foci in the mesogastric and hypogastric regions (Fig. 1). Meckel’s diverticulum scintigraphy was negative (Fig. 2).

**Figure 3:**
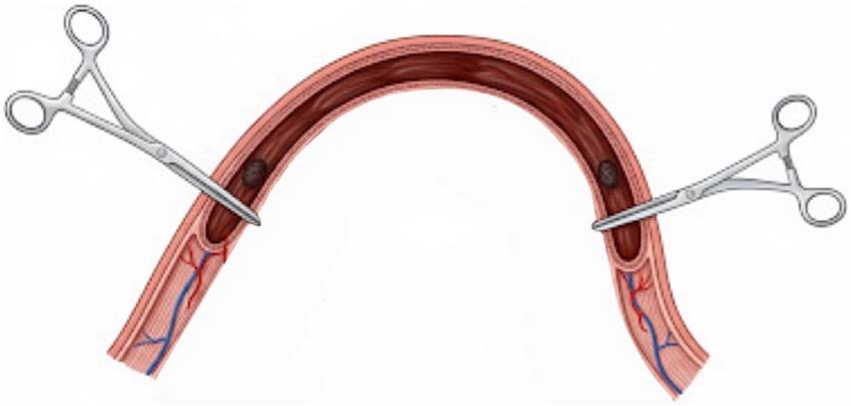
Ilustration of intraoperative isolation of jejunal nodular lesions with clamps; blood reaccumulated only in the isolated segment.

**Figure 4:**
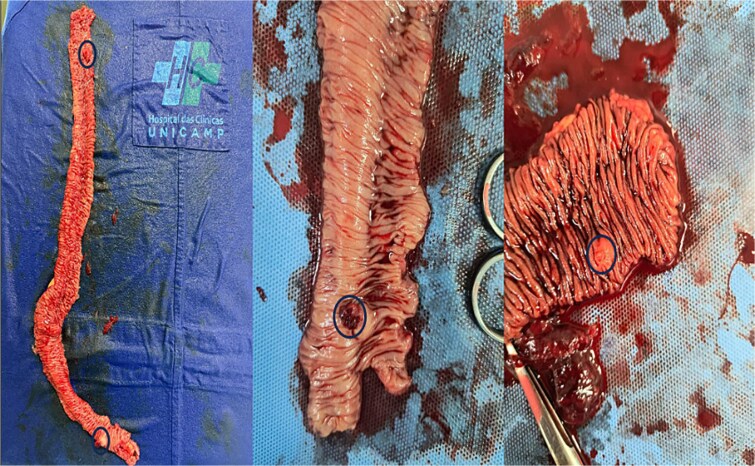
Ressected jejunal specimen with nodular lesions demarcated.

Ongoing rectal bleeding with recurrent instability required urgent laparotomy. Two intraluminal nodular lesions were found, 70 cm and 130 cm distal to the ligament of Treitz. After clamping proximally and distally to include both lesions within the isolated segment, blood reaccumulated only in this portion, confirming it as the bleeding source (Fig. 3). Segmental enterectomy (Fig. 4) with primary anastomosis was performed.

She improved clinically, was discharged from the ICU on postoperative day five, and from hospital on day seven. Outpatient follow-up showed no recurrence.

Histopathology revealed EP tissue in the submucosa and muscularis, associated with mesenchymal proliferation and fibrin deposition. Immunohistochemistry demonstrated dilated submucosal vessels with recanalizing thrombus, CD34/CD37/1A4 positivity, and no malignancy, consistent with angiodysplasia.

## Discussion

When the bleeding source is not identified by upper endoscopy or colonoscopy, the condition is classified as OGIB. A systematic approach is recommended, beginning with second-look endoscopy to exclude overlooked lesions. If these remain negative, video capsule endoscopy is the first-line investigation for the small bowel. In cases where a proximal lesion is suspected, push enteroscopy may be preferred [1, 2].

Angiodysplasia is a frequent cause of small bowel bleeding. It consists of acquired vascular malformations with dilated, thin-walled vessels in the mucosa and submucosa, prone to rupture [6]. These lesions are thought to result from chronic, intermittent venous obstruction leading to dilatation of submucosal veins and formation of fragile arteriovenous communications. They typically occur in elderly patients but may appear in younger individuals, particularly in association with chronic renal disease or aortic stenosis. Clinically, angiodysplasia may manifest as occult bleeding, iron-deficiency anemia, or overt gastrointestinal hemorrhage. Endoscopic therapy is the standard initial treatment, though rebleeding rates remain high [7]. In unstable patients or when endoscopic management fails, surgical resection remains the most definitive option [8].

EP is a rare congenital anomaly defined as pancreatic tissue located outside its normal anatomical site, without vascular or ductal continuity with the main gland [3]. Histologically, it often resides in the submucosa of the gastrointestinal tract, and its origin is attributed to aberrant migration of pancreatic primordium during embryogenesis or metaplasia of endodermal cells [4, 9]. Although most cases are asymptomatic [5, 10], EP may cause pain, obstruction, intussusception, or gastrointestinal bleeding [3, 5]. Rare associations with neoplasms have been described [11]. Bleeding is thought to occur due to secretion of pancreatic enzymes into the ectopic site, leading to mucosal inflammation, ulceration, or erosion of adjacent vessels.

In OGIB with massive bleeding and instability, angiography can identify and embolize bleeding rates as low as 1 mL/min. Where this is unavailable, alternatives include push enteroscopy or surgical exploration. OGIB presents unique challenges: the small bowel can contain extensive blood without visible lesions, especially in angiodysplasia, making localization crucial [6–8].

In this case, noninvasive imaging was inconclusive: CT angiography failed to demonstrate bleeding, likely due to low flow, while scintigraphy identified active hemorrhage but without sufficient anatomic resolution. Capsule endoscopy was planned but contraindicated due to instability. Urgent laparotomy was therefore lifesaving. Histology confirmed coexistence of EP and angiodysplasia—an exceedingly rare association that significantly complicated diagnosis.

Endoscopic evaluation remains central to OGIB management, improving diagnostic yield when feasible [1, 2, 6]. However, in unstable patients, their applicability is limited. In such scenarios, timely surgery is decisive and may be both diagnostic and therapeutic [7, 8].

### Limitations

This single retrospective case report carries the inherent limitations of the design, including lack of causal inference, limited generalizability, and susceptibility to recall and publication bias. The coexistence of EP and angiodysplasia represents an observational association only [12].

## Conclusion

EP is an uncommon cause of OGIB. When associated with angiodysplasia, diagnosis becomes particularly challenging. Management must be guided by hemodynamic stability, with surgical intervention indicated when less invasive options are inconclusive or contraindicated.
